# Is Antimicrobial Stewardship Policy Effectively Implemented in Polish Hospitals? Results from Antibiotic Consumption Surveillance before and during the COVID-19 Pandemic

**DOI:** 10.3390/antibiotics13070636

**Published:** 2024-07-10

**Authors:** Urszula Durlak, Cezary Kapturkiewicz, Anna Różańska, Mateusz Gajda, Paweł Krzyściak, Filip Kania, Jadwiga Wójkowska-Mach

**Affiliations:** 1 Students’ Scientific Group of Microbiology, Faculty of Medicine, Jagiellonian University Medical College, 31-008 Kraków, Poland; urszula.durlak@student.uj.edu.pl (U.D.); filip.kania@student.uj.edu.pl (F.K.); 2 Department of Microbiology, Faculty of Medicine, Jagiellonian University Medical College, 31-008 Kraków, Poland; mateusz14.gajda@uj.edu.pl (M.G.); pawel.krzysciak@uj.edu.pl (P.K.); jadwiga.wojkowska-mach@uj.edu.pl (J.W.-M.)

**Keywords:** antibiotic consumption, COVID-19, intensive care units, antimicrobial stewardship

## Abstract

Background: The COVID-19 pandemic posed numerous challenges to public health systems, particularly in antimicrobial stewardship. This study aimed to assess antibiotic consumption before and during the COVID-19 pandemic to evaluate the effectiveness of the implemented antimicrobial stewardship program. Methods: This retrospective study was carried out at the University Hospital in Krakow, Poland, between 1 January 2019 and 31 December 2020. A total of 80,639 patients were enrolled. Antibiotic usage was measured as the percentage of patients receiving antibiotics and the number of days of therapy (DOTs). The World Health Organization (WHO) methodology and Anatomical Therapeutic Chemical (ATC) codes and AWaRe classification were utilized. The analyzed ATC antibiotic groups included penicillins (J01CA, J01CE, J01CF, J01CR, excluding piperacillin/tazobactam), piperacillin with tazobactam-beta-lactamase inhibitor (J01CR05), third- and fourth-generation cephalosporins (J01DD, J01DE), carbapenems (J01DH), macrolides (J01FA), fluoroquinolones (J01M), colistin (J01XB01), metronidazole (J01XD01) and others (J01DF, J01DI, J01E, J01G, J01XA, J01A). In the AWaRe classification, Access, Watch and Reserve groups of antibiotics were included. Results: In 2020, 79.2% of COVID-19 patients and 40.1% of non-COVID-19 patients were treated with antibiotics, compared to 28.8% in 2019. Also, in 2020, the antibiotic consumption in non-ICU COVID-19 patients was twice as high as in non-COVID-19 patients: 50.9 vs. 38.5 DOTs/100 patient days (pds). Conversely, in the ICU, antibiotic consumption in COVID-19 patients was 112.1 DOTs/100 pds compared to 248.9 DOTs/100 pds in non-COVID-19 patients. Significant increases were observed in the usage of third- and fourth-generation cephalosporins in 2020. The analysis according to the AWaRe system revealed the highest usage of the Watch group—ranging from 61.9% to 78.7%—and very high usage of the Reserve group—from 5.8% to 11.1%—in non COVID-19 and COVID-19 patients, respectively. Conclusions: Our findings highlight substantial issues with antibiotic use both before and during the COVID-19 pandemic. The results underscore the urgent need for improved antimicrobial stewardship policy implementation.

## 1. Introduction

Coronavirus disease 2019 (COVID-19) is a contagious disease caused by severe acute respiratory syndrome coronavirus 2 (SARS-CoV-2). The COVID-19 epidemic began on 17 November 2019, in China [1]. Its rapid spread posed a serious threat to international public health, leading the World Health Organization (WHO) to declare it a pandemic on 11 March 2020.

The disease is characterized by a range of symptoms, including fever, cough, hyposmia, sputum production, chest tightness, dyspnea and myalgia [2]. In severe cases, it can lead to complications such as acute respiratory distress syndrome, septic shock and renal failure [3]. Despite this, many cases were asymptomatic, complicating prevention and control efforts. During the first half of 2020, at the University Hospital in Krakow (UHK), the severity of COVID-19, according to WHO classification, was categorized as mild in 67% of patients, moderate in 18% and severe or critical in 15% [4]. However, initial variants such as Alpha, Beta, Gamma and Delta that emerged in 2020, and dominated globally for most of 2021, were replaced in late 2021 by the Omicron variant, which exhibited much higher transmissibility and transmission efficiency than all previous variants [5].

The leading therapeutic agents recommended against COVID-19 from March to June 2020 included azithromycin, dexamethasone and remdesivir, as per WHO guidelines [6]. Strictly taking one of these into consideration, azithromycin—mentioned in detailed analyses by some authors—shows no effect on reducing patient mortality or improving the general clinical condition [7]. From October 2021, the European Medicines Agency (EMA) also recommended tocilizumab for COVID-19 treatment [8]. In addition to antiviral therapies, the 2020 treatment regimen for COVID-19 incorporated supportive treatments, primarily the use of steroids [6,9].

In the early stages of the pandemic, the absence of approved targeted treatments posed significant challenges for managing COVID-19. The WHO advises against antibiotic therapy or prophylaxis for patients with mild to moderate COVID-19, except in cases where there is a clinical suspicion of bacterial coinfection. However, for patients with severe COVID-19, the WHO recommends the prompt initiation of empiric antimicrobial therapy. This approach targets likely pathogens and is informed by clinical judgment, patient-specific factors, and local epidemiological data [10].

Rawson et al. reported widespread use of broad-spectrum antibacterials in hospitals, despite limited evidence of bacterial coinfection. Their study indicated that only approximately 8% of patients hospitalized due to COVID-19 experienced bacterial or fungal coinfections during admission, compared to 11% in non-COVID-19 cases. Additionally, the study revealed that over 70% of COVID-19 patients received antimicrobial therapy, with no antimicrobial stewardship interventions documented [11]. Data published after the pandemic or from its late stage emphasize a lack of clinical benefits and even indicate a possible increase in resistance as a result of incorrectly administered therapy [12].

It should be noted that the use of antibiotics can have specific adverse side effects, including allergic reactions and dose-related hematological, gastrointestinal, renal or hepatic complications that directly impact the patient [10]. Additionally, the inappropriate use of antibiotics is a significant contributing factor to the growing problem of multidrug-resistant microorganisms (MDROs) [13,14]. MDROs are now considered to pose a serious threat to human health worldwide. According to WHO estimates, in 2019 alone, MDROs were responsible for 4.95 million deaths globally and 67.7 cases per 100,000 population in Central Europe annually [14]. The situation in Poland is particularly alarming. For instance, at the UHK, in 2021, the prevalence of extensively drug-resistant organisms was 22.6% in intensive care units (ICUs) and 14.8% in non-ICUs among all isolates in healthcare-associated infections [15]. MDR, the leading cause of morbidity and mortality from previously treatable infections, is therefore believed to be primarily related to the excessive or inappropriate use of antibiotics [16,17].

The objective of the study was to assess antibiotic consumption trends before and during the COVID-19 pandemic as an indicator of the effectiveness of implementing an antimicrobial stewardship program.

## 2. Results

**Figure 1:**
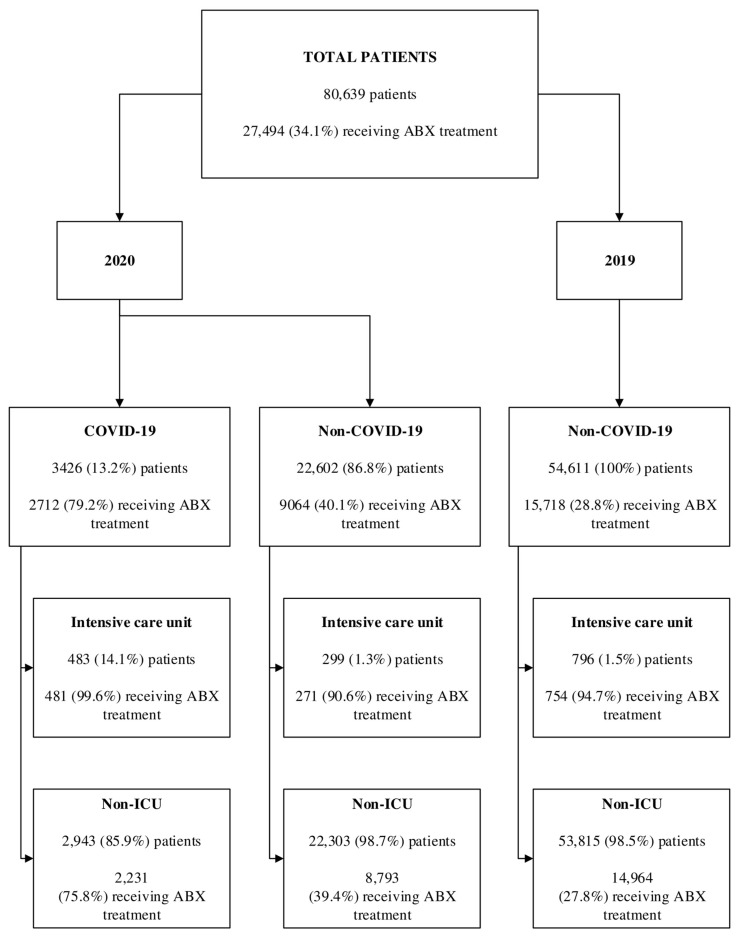
Antibiotic consumption at University Hospital in Krakow, stratified by year (2019 and 2020), COVID-19 status and hospitalization setting (ICU vs. non-ICU).

Out of the total 80,639 adult patients, 54,611 were admitted to the hospital in 2019 and 26,028 in 2020. In 2019, 34.1% of the patients received antibiotic treatment. In 2020, 40.1% of non-COVID-19 and 79.2% of COVID-19 patients were treated with antibiotics. In the ICU, there were 796 patients in 2019 and 782 patients in 2020, with 483 (61.7%) diagnosed with COVID-19. The proportion of ICU patients receiving antibiotics was 94.7%, 90.6% and 99.6%, respectively (Figure 1).

### 2.1. Non-ICU Patients

The demographic characteristics of the patient populations differed between the study years. In 2020, the median patient age was higher compared to 2019, and there was a higher proportion of male patients among those diagnosed with COVID-19 (Table 1). The median length of hospital stay also showed a significant increase in 2020, with non-COVID-19 patients having a median stay of 4.0 days and COVID-19 patients having a median stay of 16.0 days, compared to a median stay of 3.0 days in 2019. Furthermore, the use of steroid therapy increased from 16.8% in 2019 to 26.0% in 2020 among non-COVID-19 patients and 48.45% among COVID-19 patients (Table 1).

### 2.2. ICU Patients

Among ICU patients, the median age decreased in 2020 for non-COVID-19 patients but was insignificantly higher for the COVID-19 population. However, the proportion of male patients was significantly higher only in the COVID-19 patient group, with an increase of approximately 7 percentage points. The median length of stay increased significantly only for COVID-19 patients. The proportion of patients who stayed for 3 days or longer did not differ significantly across the three groups. Nearly every COVID-19 patient (93.2%) received some form of steroid treatment, and the median IV steroid DOTs increased significantly, from 1.0 day in 2019 to 10.0 days in 2020 for COVID-19 patients (Table 1).

### 2.3. Antibiotic Consumption

In 2019, antibiotic therapy accounted for a total of 114,177 days in the non-ICU setting, equating to 23.9 DOT/100 pds, and 24,412 days in the ICU, corresponding to 163.6 DOT/100 pds. In 2020, the antibiotic consumption in the non-ICU setting was 26.7 DOT/100 pds for non-COVID-19 patients and 47.8 DOT/100 pds for COVID-19 patients. In the ICU, antibiotic consumption was 217.2 DOT/100 pds for non-COVID-19 patients and 110.6 DOT/100 pds for COVID-19 patients (Table 2).

In the non-ICU settings in 2020, the most commonly used antibiotics were third-and fourth-generation cephalosporins (3GC and 4GC). Their usage increased significantly from 2.9 DOT/100 pds in 2019 to 24.1 DOT/100 pds in COVID-19 patients (OR 7.85 95% CI (7.63–8.07)) and 7.3 DOT/100 pds in non-COVID-19 patients (OR 2.71 95%CI (2.63–2.78)) (Table 2).

Among ICU patients, carbapenems were the most frequently used antibiotics in both years of the study. In 2019, the usage was 59.9 DOT/100 pds, which increased significantly in 2020 to 61.8 DOT/100 pds for non-COVID-19 patients (OR 0.75 95% CI (0.71–0.79)). However, the usage decreased to 30.5 DOT/100 pds in COVID-19 patients (OR 0.8). Notably, the proportion of carbapenem usage among all antibiotics decreased significantly in 2020 in both groups, from 30.48% in 2019 to 24.84% in non-COVID-19 patients and 25.42% in COVID-19 patients (OR 0.75 95% CI (0.71–0.79)). Additionally, there was a notable increase in the use of 3GC and 4GC in both studied populations, as well as an increased usage of colistin in COVID-19 patients (OR 1.5) (Table 3).

**Figure 2:**
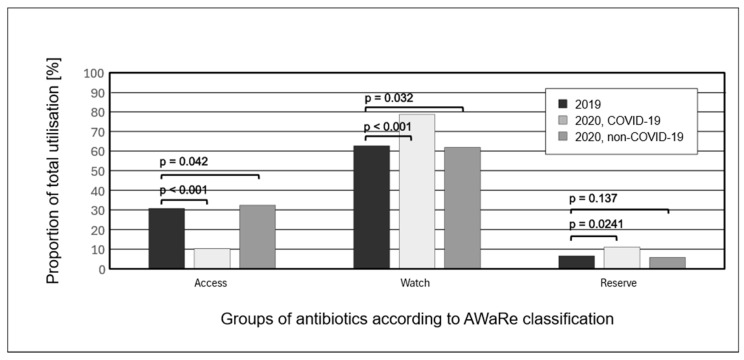
Distribution of antibiotic groups according to AWaRe (Access, Watch, Reserve) classification in studied populations.

Comparison of utilization of antibiotics according to AWaRe stratification in analyzed periods revealed a significant increase in the Watch group during the pandemic, both in COVID-19 (*p* < 0.001) and non-COVID-19 patients (*p* = 0.032): from 62.7% in 2019 to 78.7% and 61.9%, respectively, in 2020. There are no significant differences for the proportion of the Reserve group, which was 6.6% in 2019 and in 2020—11.1% in COVID-19 and 5.8% in non-COVID-19 patients (Figure 2).

## 3. Discussion

This study demonstrated an increase in antibiotic consumption during the initial phase of the COVID-19 pandemic in 2020 compared to 2019, irrespective of the reason for hospitalization. This increase was observed both in the proportion of patients receiving antibiotics and in the measure of DOT/100 pds, with some antibiotics showing a marked predominance.

In 2020, approximately 40% of non-COVID-19 patients in non-ICU settings received antibiotics, compared to nearly 30% in 2019. Although the overall usage level did not change significantly, there was a notable increase in the duration of antibiotic therapy in in both years, suggesting alterations in treatment patterns, particularly in the distribution of different antibiotic groups. Antibiotics were administered to 75.8% of COVID-19 patients in 2020, indicating a common practice of using antibiotics, possibly for prophylaxis against secondary bacterial infections rather than for treatment. Such an increase in the use of antibiotics in our study was not an isolated incident. A multi-center meta-analysis conducted in April 2021, primarily based in China, demonstrated a 74.8% occurrence of antibiotic therapy in inpatient settings [12], with ICU consumption rates reaching up to 86.4% [18]. However, a study by Wang et al. reported a decrease in overall antibiotic consumption in selected township hospitals by 32.04% in 2020 and 16.69% in 2021, compared to 2019 [19].

The emergence of COVID-19 introduced numerous uncertainties regarding the progression of the disease in individual patients and potential complications, possibly leading to various practices without strong scientific evidence. This increase in antibiotic consumption highlights potential gaps in the scientific basis for antibiotic therapy in Poland and the effectiveness of antimicrobial stewardship programs (ASPs). This is extremely important in intensive care units. Despite reports from the ESAC-Net ECDC program indicating that average antibiotic consumption in Polish hospitals was slightly lower than the European average in 2019 and 2020 [20], reliable assessment remains challenging using the DDD per 1000 inhabitants per day metric. Studies targeted to analyze antibiotic consumption in a more detailed way at the hospital level consistently show high antibiotic usage in Polish hospitals, especially in ICUs. This observation is correlating with high rates of MDR strains isolated from invasive infections [21] and some of the highest CDI incidence rates [22] in Europe. Evaluations of antibiotic consumption typically use defined daily doses (DDDs) and days of therapy (DOTs). In hospital settings, it is crucial to relate these metrics to admissions or person days and consider the specific characteristics of different units, particularly ICUs, where antibiotic consumption is highest. For instance, Polish ICU studies by Ziółkowski et al. and Trejnowska et al. reported average antibiotic consumption approximately 30% higher than in Sweden and Germany [23,24,25,26] and three times higher than in Indian ICUs, as reported by Singh et al. [27]. Furthermore, Polish ICU antibiotic consumption was several times higher than in Saudi Arabia, as reported by Balkhy et al. [28]. Singh et al. documented antibiotic consumption ranging from 44.01 to 52.14 DOTs/100 pds (in various months of the study period) for empirical therapy and 32.32 to 51.84 DOTs/100 pds for targeted therapy. In our study, ICU patients had an average consumption of 196.7 DOTs/100 pds in 2019. In 2020, ICU patients with COVID-19 had an average consumption of 112.1 DOTs/100 pds, while non-COVID-19 ICU patients had 248.9 DOTs/100 pds. Balkhy et al. reported an average of 83.65 DOTs/100 pds [28].

These discrepancies indicate divergent approaches to antibiotic use, not only in ICU settings but also across various treatment regimens. The 2017 ECDC study on eight European countries reported carbapenem consumption ranging from 2.9 to 19.7 DOTs/100 pds, 3GC and 4GC: 0.0 to 18.2 DOTs/100 pds and fluoroquinolones: 3.5 to 15.5 DOTs/100 pds [28]. In contrast, our study found consumption among non-COVID-19 patients in 2019 and 2020 to be significantly higher: 59.9, 61.8 for carbapenems and 30.5, 12.8 for 3GC and 4GC, and 44.8, 18.0 for fluoroquinolones. The markedly higher consumption of carbapenems and fluoroquinolones, which belong to the WHO AWaRe ‘Watch’ group, is concerning. Unfortunately, this does not only refer to fluoroquinolones—our analysis according to the AWaRe system revealed use of the Reserve group ranging from around 6 to 11% and of the Watch group about 60% in patients without COVID-19 and almost 80% in those with COVID-19. This is undoubtedly alarming and opposite to the expected pattern. Proud et al., in Scottish hospitals during the COVID-19 pandemic, reported usage of Access antibiotics of around 60%, Watch antibiotics of around 40% and Reserve antibiotics between 1.4 and 2.1% [29].

Pre-pandemic studies suggest that many clinicians are hesitant to discontinue antimicrobial treatment once initiated, and antibiotics are often used as prophylaxis in ICU patients, especially those at high risk of infection [30]. Although COVID-19 may elevate the risk of secondary bacterial infections, multiple studies have shown a significant disparity between the actual incidence of such infections and the administration of antimicrobial treatment [12,18]. Martin-Loeches et al., in a meta-analysis of inpatient secondary infection rates conducted throughout 2020, showed major discrepancies between the rate of secondary infections and antibiotic usage. The rate of antibiotic prescriptions ranged from 60% to 100% in some studies, which is significantly higher than necessary for the observed incidence of secondary infection, whereby bacterial infections occurred in 16% of patients (with figures ranging from 4.8% to 42.8%) and secondary fungal infections found in 6.3% of patients (with figures ranging from 0.9% to 33.3%) [31]. A meta-analysis published in June 2021 exhibited similar trends. In Europe, antibiotic therapy was use in 60% of COVID-19 patients, but secondary bacterial infections were present in only about 4%. However, the cited studies had significant sample heterogeneity, which may affect data precision [32].

Given the increased prevalence of antibiotic treatment during the early stages of the pandemic, we may face a decrease in antimicrobial treatment’s effectiveness due to rising antibiotic resistance.

High rates of antibiotic prescriptions have been a persistent problem throughout the pandemic. Multicenter retrospective studies from Europe are in line with those findings, confirming a lack of clinical effect and a risk associated with the use of antibiotics in the early phase of the pandemic [33]. Such observations indicate that antimicrobial stewardship is not effectively implemented.

The other issue is the diversity of antibiotic treatment. The classes of antibiotics used vary widely across countries. In China, fluoroquinolones are the most commonly used, followed by β-lactams + BLI and cephalosporins. In North America, macrolides are the most frequently used, followed by β-lactams + BLI, cephalosporins, and β-lactams. Europe displays different trends, with the most frequently used antibiotics being β-lactams, macrolides, cephalosporins, and β-lactams + BLI, respectively [12]. Thus, for everyday practice, establishment and effective implementation of antimicrobial stewardship is crucial. One element of such programs is to analyze the level and structure of antimicrobial consumption in the context of specific patient populations [33].

Current WHO guidelines for COVID-19 treatment do not recommend the routine use of antibiotics, except in cases of secondary bacterial infection [34]. Considering these findings, the medical community should reconsider the use of antibiotics if a new viral pandemic occurs. In future pandemics, despite utilizing antibiotics for their other mechanisms of action, it is crucial to incorporate antibiotic stewardship and remember their original intended use.

There is no effectively implemented antibiotic stewardship without continuously measuring the outcomes and providing feedback to main groups of interest [35]. Assessing the consumption of antibiotics is one element of such programs and should be conducted routinely, both at the hospital level and regional or country level as well. This is necessary for benchmarking, which is a first step in the optimalization and improvement of antibiotic usage in patients. But, at a higher level, One Health data on antibiotic usage and patterns are extremely valuable in order to create complex policies and actions for humans and the whole environment [36].

## 4. Materials and Methods

### 4.1. Study Design and Study Population

This retrospective before-and-after study was conducted at the University Hospital in Krakow, Poland (UHK), a tertiary care facility with 1310 acute care beds located in southern Poland. Between March and September 2020, the UHK was exclusively designated for the treatment of COVID-19 patients. Subsequently, from October 2020 to March 2022, these patients were hospitalized in a separate dedicated building, which housed 200 adult beds, including 50 intensive care beds. Patients included in the study were diagnosed with COVID-19 following WHO guidelines, utilizing RT-PCR methods for SARS-CoV-2 detection (COBAS 6800, Roche or GeneXpert System, Cepheid, Sunnyvale, CA, USA, in CITO mode). Inclusion criteria encompassed all patients diagnosed with COVID-19, irrespective of their primary complaints or presenting symptoms. The study analyzed data from 80,638 adult patients (including pregnant women) admitted to the UHK between 1 January 2019 and 31 December 2020, excluding those with hospital stays of one day. For the analysis of antibiotic use, patients administered cefazolin for surgical prophylaxis, the most frequently used antibiotic for this purpose, were excluded.

We collected data on patient’s age, sex, length of stay (LOS) expressed in days, use of antibiotic and steroid therapy (as percentage and days of therapy), ICU admission, and PCR-confirmed COVID-19 status. The data were derived from the hospital electronic data base. All data were anonymized prior to inclusion in the study and analysis. The study protocol was approved by the Bioethics Committee of the Jagiellonian University (protocol code 1072.6120.2.2021, date of approval: 20 January 2021). The survey was carried out in accordance with the principles contained in the Helsinki Declaration as revised in 2013.

### 4.2. Antibiotic Consumption Measure

Two WHO approaches for antibiotic consumption measure were used—the Anatomical Therapeutic Chemical (ATC) classification system and a categorization including three groups: Access, Watch and Reserve (AWaRe). The WHO ATC system focused on two main groups: H02 for corticosteroids for systemic use and J01 for antibacterials for systemic use. Antibacterials were categorized based on their mode of action and chemical properties [37]. The following antibiotic groups were distinguished: penicillins (J01CA, J01CE, J01CF, J01CR, excluding piperacillin/tazobactam), piperacillin with tazobactam-beta-lactamase inhibitor (J01CR05), third- and fourth-generation cephalosporins (J01DD, J01DE), carbapenems (J01DH), macrolides (J01FA), fluoroquinolones (J01M), colistin (J01XB01), metronidazole (J01XD01) and other classes (J01DF, J01DI, J01E, J01G, J01XA, J01A).

The AWaRe system is a compiled list of 180 commonly used antibiotics in three categories. The Access group consists of first-choice agents for common infections, generally with a narrower spectrum of action. The Watch group contains antibiotics more commonly used as second-line treatments or for treating resistant organisms. The Reserve group contains antibiotics considered drugs of last resort, where no other options are available [38].

We utilized the following measures to characterize antibiotic consumption in the studied population: antibiotic days of therapy (DOTs) and DOTs per 100 patient days (pds). The latter represents the total number of days a specific antibiotic was administered against the total number of pds.

### 4.3. Statistical Analysis

Statistical analysis was conducted using the R language and environment for statistical computing, version 4.2.3 (released on 15 March 2023, R Core Team, 2023), within the RStudio environment (version 2023.03.0). Given that most of the data did not follow a normal distribution, we applied non-parametric tests. Specifically, we used the Wilcoxon rank sum test with continuity correction (U–Mann test) to compare two independent variables. For categorical data, we employed Pearson’s Chi-squared test with Yates’ continuity correction and Fisher’s exact test to calculate odds ratios. For analysis of antibiotic utilization according to AWaRe classification, the Bonferroni correction was used. In all analyses, a *p*-value of ≤0.05 was considered statistically significant.

## 5. Limitations

A primary limitation of this study is its temporal scope, which only includes the early waves of the COVID-19 pandemic and excludes data from 2021 to 2022. This restricts our understanding of whether the observed trends in antibiotic consumption persist post-pandemic and their long-term implications. The absence of clear treatment guidelines during the initial stages of the pandemic likely contributed to increased antibiotic use. However, this does not fully account for the unwarranted prophylactic use of antibiotics observed in clinical practice. Thus, comparing our findings with pre-pandemic data is crucial for understanding antibiotic consumption trends and assessing antimicrobial stewardship during the pandemic.

## 6. Conclusions

Our findings reveal a high level of antibiotic consumption both before and during the COVID-19 pandemic. Another disturbing observation is the high percentage of antibiotics from the Watch group, together with the substantial use of those from the Reserve group (WHO AWaRe classification). Alongside other reports from Poland, our results suggest that antimicrobial stewardship in Polish hospitals is suboptimal and necessitates substantial improvements.

## Figures and Tables

**Table 1:** Demographic characteristics, LOS and steroid usage in patients at University Hospital in Krakow for the years 2019 and 2020, according to the type of ward.

Hospitalization		2019	2020	
			Non-COVID-19 Patients	COVID-19 Patients
**Patients in Non-Intensive Care Unit**		**N = 53,815**	**N = 22,303**	**N = 2943**
Age [years]	Median (Q1; Q3)	51.00 (32.00–68.00)	57.00 (36.00–70.00)	63.00 (50.00–75.00)
			*p* < 0.001	*p* < 0.001
Male, yes	N (%)	23,284 (43.27)	9238 (41.42)	1543 (52.43)
			*p* < 0.001	*p* < 0.001
Inpatient care > 48 h, yes	N (%)	30,785 (57.21)	16,828 (75.45)	2892 (98.27)
			*p* < 0.001	*p* < 0.001
Length of stay [days]	Median (Q1; Q3)	3.00 (1.00–7.00)	4.00 (2.00–8.00)	16.00 (11.00–24.00)
			*p* < 0.001	*p* < 0.001
Steroid use	N (%)	9029 (16.78)	5804 (26.02)	1426 (48.45)
			*p* < 0.001	*p* < 0.001
IV steroid therapy [DOT]	Median (Q1; Q3)	0.00 (0.0–0.0)	0.00 (0.00–1.00)	0.00 (0.00–8.00)
			*p* < 0.001	*p* < 0.001
**Patients in Intensive Care Unit**		**N = 796**	**N = 299**	**N = 483**
Age [years]	Median (Q1; Q3)	66.00 (54.00–76.00)	64.00 (49.00–72.50)	66.00 (57.00–74.00)
			*p* = 0.002	*p* = 0.692
Male, yes	N (%)	479 (60.18)	184 (61.54)	341 (70.60)
			*p* = 0.733	*p* < 0.001
Inpatient care > 48 h, yes	N (%)	742 (93.21)	267 (89.30)	469 (97.10)
			*p* = 0.043	*p* = 0.002
Length of stay [days]	Median (Q1; Q3)	21.00 (8.00–39.00)	13.00 (3.50–28.50)	21.00 (12.00–35.00)
			*p* < 0.001	*p* = 0.278
Intensive care LOS [days]	Median (Q1; Q3)	9.00 (3.00–20.00)	5.00 (2.00–15.50)	11.00 (6.00–18.00)
			*p* < 0.001	*p* < 0.001
Steroid use	N (%)	508 (63.82)	179 (59.87)	450 (93.17)
			*p* = 0.256	*p* < 0.001
IV steroid therapy [DOT]	Median (Q1; Q3)	1.00 (0.00–5.00)	1.00 (0.00–3.00)	10.00 (5.00–13.00)
			*p* = 0.072	*p* < 0.001

**Table 2:** Antibiotic usage among non-ICU patients at University Hospital in Krakow in the years 2019 and 2020.

Non-ICU Patients	2019, DOT		2020 Non-COVID-19 Patients, DOT			2020 COVID-19 Patients, DOT		
ABX Group	N (%)	Per 100 pds	N (%)	Per 100 pds	OR (95%CI), *p*-Value *	N (%)	Per 100 pds	OR (95%CI), *p*-Value *
Penicillins (without PIP/TAZ)	8243 (7.22)	2.0	4594 (7.66)	2.4	1.07 (1.03–1.11), *p* < 0.001	570 (1.62)	0.8	0.21 (0.19–0.23), *p* < 0.001
PIP/TAZ	3426 (3.00)	0.8	2520 (4.20)	1.3	1.42 (1.34–1.49), *p* < 0.001	481 (1.37)	0.7	0.45 (0.41–0.49), *p* < 0.001
3GC and 4GC	11,720 (10.26)	2.9	14,177 (23.63)	7.3	2.71 (2.63–2.78), *p* < 0.001	16 632 (47.30)	24.1	7.85 (7.63–8.07), *p* < 0.001
Carbapenems	20,444 (17.91)	5.1	8999 (15.00)	4.6	0.81 (0.79–0.83), *p* < 0.001	6311 (17.95)	9.1	1.00 (0.97–1.03), *p* = 0.86
Macrolides	2084 (1.83)	0.5	597 (1.00)	0.3	0.54 (0.49–0.59), *p* < 0.001	794 (2.26)	1.2	1.24 (1.14–1.35), *p* < 0.001
Fluoroquinolones	33,193 (29.07)	8.2	13,779 (22.97)	7.1	0.73 (0.71–0.74), *p* < 0.001	6225 (17.70)	9.0	0.52 (0.51–0.54), *p* < 0.001
Colistin	1245 (1.09)	0.3	637 (1.06)	0.3	0.97 (0.88–1.07), *p* = 0.59	488 (1.39)	0.7	1.28 (1.15–1.42), *p* < 0.001
Metronidazole	16,114 (14.11)	4.0	6853 (11.42)	3.5	0.78 (0.76–0.81), *p* < 0.001	1483 (4.22)	2.1	0.27 (0.26–0.28), *p* < 0.001
Others	17,708 (15.51)	4.4	7830 (13.05)	4.0	0.82 (0.79–0.84), *p* < 0.001	2182 (6.20)	3.2	0.36 (0.34–0.38), *p* < 0.001
Total	114,177 (100)	28.2	59,986 (100)	30.8	--	35,166 (100)	50.9	--

* assessed with Fisher’s Exact Test for Count Data, 3GC and 4GC—3rd and 4th generation cephalosporins; ABX, antibiotics; DOTs—days of therapy; pds—patient days; PIP/TAZ—piperacillin + tazobactam.

**Table 3:** Antibiotic usage among ICU patients at University Hospital in Krakow in 2019 and 2020.

ICU Patients Only	2019, DOT		2020 Non-COVID-19 Patients, DOT			2020 COVID-19, DOT		
ABX Group	N (%)	Per 100 pds	N (%)	Per 100 pds	OR (95%CI), *p*-Value *	N (%)	Per 100 pds	OR (95%CI), *p*-Value *
Penicillins (without PIP/TAZ)	985 (4.03)	7.9	163 (1.93)	4.8	0.47 (0.39–0.55), *p* < 0.001	665 (7.66)	9.2	1.98 (1.78–2.18), *p* < 0.001
PIP/TAZ	210 (0.86)	1.7	153 (1.81)	4.5	5.05 (4.38–5.85), *p* < 0.001	120 (1.38)	1.7	1.61 (1.36–1.89), *p* < 0.001
3GC and 4GC	1587 (6.50)	12.8	1 523 (18.00)	44.8	4.45 (4.22–4.70), *p* < 0.001	1 304 (15.00)	18.0	2.54 (2.35–2.75), *p* < 0.001
Carbapenems	7440 (30.48)	59.9	2 102 (24.84)	61.8	0.75 (0.71–0.79), *p* < 0.001	2 209 (25.42)	30.5	0.78 (0.73–0.82), *p* < 0.001
Macrolides	38 (0.16)	0.3	16 (0.19)	0.5	1.21 (0.63–2.23), *p* = 0.53	36 (0.41)	0.5	2.67 (1.64–4.33), *p* < 0.001
Fluoroquinolones	4419 (18.10)	35.6	1871 (22.11)	55.0	1.28 (1.21–1.37), *p* < 0.001	2 063 (23.74)	28.5	1.41 (1.33–1.50), *p* < 0.001
Colistin	2690 (11.02)	21.7	561 (6.63)	16.5	0.57 (0.52–0.63), *p* < 0.001	1 342 (15.44)	18.5	1.47 (1.37–1.58), *p* < 0.001
Metronidazole	2951 (12.09)	23.8	999 (11.81)	29.4	0.97 (0.90–1.05), *p* = 0.50	273 (3.14)	3.8	0.24 (0.21–0.27), *p* < 0.001
Others	4092 (16.76)	33.0	1 074 (12.69)	31.6	0.72 (0.67–0.78), *p* < 0.001	678 (7.80)	9.4	0.42 (0.39–0.46), *p* < 0.001
Total	24,412 (100)	196.7	8462 (100)	248.9	--	8690 (100)	112.1	--

* assessed with Fisher’s Exact Test for Count Data, 3GC and 4GC—third- and fourth-generation cephalosporins; ABX, antibiotics; DOTs—days of therapy; pds—patient days; PIP/TAZ—piperacillin + tazobactam.

## Data Availability

The datasets generated or analyzed during this study are available and can be obtained upon request from the corresponding author on reasonable inquiry.

## Abbreviations

Anatomical Therapeutic Chemical Code (ATC code); Coronavirus disease 2019 (COVID-19); days of therapy (DOTs); European Medicines Agency (EMA); Intensive Care Unit (ICU); length of stay (LOS); patient days (pds); Severe Acute Respiratory Syndrome Coronavirus 2 (SARS-CoV-2); University Hospital in Krakow (UHK); World Health Organization (WHO).
